# Effect of Lactic Acid Fermentation on Quinoa Characteristics and Quality of Quinoa-Wheat Composite Bread

**DOI:** 10.3390/foods10010171

**Published:** 2021-01-16

**Authors:** Dalia Cizeikiene, Ieva Gaide, Loreta Basinskiene

**Affiliations:** Department of Food Science and Technology, Kaunas University of Technology, Radvilenu Rd. 19, 50254 Kaunas, Lithuania; dalia.cizeikiene@ktu.lt (D.C.); ieva.gaide@ktu.edu (I.G.)

**Keywords:** quinoa, *Lactobacillus*, fermentation, quinoa-wheat composite bread, protein fractions, hydrolysates, antioxidant activity

## Abstract

The application of selected starter cultures with specific properties for fermentation may determine steady lactic acid bacteria (LAB) variety and the characteristics of fermented products that influence nutritional value, the composition of biologically active compounds and quality. The aim of this research was to evaluate the influence of different LAB on the biochemical characteristics of fermented quinoa. Moreover, total phenolic content (TPC), and the antimicrobial and antioxidant activities of protein fractions isolated from quinoa previously fermented with LAB were investigated. Quinoa additives, including quinoa fermented with *Lactobacillus brevis*, were incorporated in a wheat bread recipe to make nutritionally fortified quinoa-wheat composite bread. The results confirmed that *L. plantarum*, *L. brevis*, and *L. acidophilus* were well adapted in quinoa medium, confirming its suitability for fermentation. LAB strains influenced the acidity, L/D-lactic acid content, enzyme activity, TPC and antioxidant activity of fermented quinoa. The maximum phytase activity was determined in quinoa fermented with *L. brevis*. The results obtained from the ABTS radical scavenging assay of protein fractions confirmed the influence of LAB strain on the antioxidant activity of protein fractions. The addition of 5 and 10% of quinoa fermented with *L. brevis* did not affect the total titratable acidity of wheat bread, while 10% of fermented quinoa with *L. brevis* resulted in a higher specific volume. Fermented quinoa additives increased the overall acceptability of bread compared with unfermented seed additives.

## 1. Introduction

Quinoa (*Chenopodium quinoa* Willd.) is a well-known industrially valuable pseudocereal due to its potential application as an important source of nutrients, fibres, and bioactive compounds in particular [1,2]. Quinoa is considered plant-based food product suitable for all people, mainly for health-conscious consumers, vegetarians, and athletes, specifically because of the balanced amino acid profile and good balance between components such as carbohydrates, lipids, proteins, as well as for consumers with celiac disease [3]. Recently, the application of quinoa in bread and sourdough production using selected LAB strains has attracted much interest [4,5,6]. LAB play a significant role in food fermentations, where they contribute to the development of the wanted technological properties, sensory characteristics and microbiological safety in the food products. The application of selected LAB as starters permits the quality of bread products and their nutritional value to be improved through metabolic activity [7,8] and the release of peptides with antioxidant activity [9,10].

Recently, the number of investigations in the arena of bioactive proteins and peptides from cereals and pseudocereals has increased [11,12]. Until now, the focus has been on the technological and functional properties, protein digestibility, essential amino acid composition of quinoa, the application of protein isolates from quinoa as a functional component in the food industry [13]. Much attention is paid to plant proteins and their hydrolysates [14,15,16]. Bioactive proteins and peptides are natural compounds present in plant and animal products that possess the ability to improve certain health functions once present in the human body [11]. Low molecular weight proteins or peptides from foods are derived by the activity of enzymes; when they get to the human body, they can act as modulators of particular physiological processes [17]. The physiological effects of proteins and peptides have been demonstrated by their action on the regulation of elevated blood pressure, modulation of the immune system, improving the inflammatory and neuroinflammatory states, lowering cholesterol and triglyceride levels, stimulating the nervous system, improving the transport and absorption of minerals, and affecting antioxidant and antimicrobial activities [18]. In view of the expected adverse effects of synthetic antioxidants on human health, antioxidants from natural sources have attracted considerable interest. Protein fractions such as albumin, globulin, glutelin and prolamine are classified by solubility according to Osborne’s classification. Protein fractions with antioxidant activity may also be used in food production as a supplement or functional ingredient to increase the shelf life, nutritional value or functional properties of the product. Recently, protein fractions and their hydrolysates from plant, seeds showing antioxidant activity have gained great attention [19,20,21].

To date, the characterisation of the bioactive quinoa protein hydrolysates using proteolytic LAB remains widely underinvestigated. Few studies have demonstrated that quinoa protein hydrolysates obtained from enzymatic action showed an antioxidant effect [16]. Coda et al. [9] isolated peptides with antioxidant properties from cereal flours previously fermented with LAB. However, to the best of our knowledge, the characterisation of the antimicrobial and antioxidant activity of different protein fractions isolated from quinoa fermented with LAB is very limited. Rizzello et al. [10] revealed the capacity of some LAB to release peptides with antioxidant activity in quinoa flour. The fermentation of proteins with LAB can influence protein hydrolysis caused by the proteolytic activity of LAB, and release numerous peptides during the fermentation of proteins [22]. This protease activity makes free amino acids necessary for bacterial multiplication and also results in a wide variety of peptides characterised with biological properties [23,24]. These bioactive peptides are interesting from a nutritional and healthcare viewpoint. The production of bioactive compounds by LAB species is relatively inexpensive, compared to the use of purified commercial enzymes; moreover, the application of newly isolated LAB has a greater potential to produce new peptides [25], meaning that new bioactive compounds with specific biological properties could be obtained. The fermentation of quinoa flour using LAB could be an effective strategy for the production and valorisation of new bioactive peptides with specific biological properties such as antimicrobial and antioxidant activities. Each LAB species and strain exhibits different proteinase activities, leading to a large variety of proteolysis products [25]. These findings motivated the evaluation of other LAB as a starter for quinoa fermentation for the production of novel and healthy baked goods; in particular, newly isolated strains from traditional rye sourdough and even from the human intestine are of great interest.

Moreover, strains that produce phytases could improve mineral bioavailability from baked goods [26,27]. Therefore, those strains are preferable as starters for sourdough production. Quinoa fermented with LAB could be applied as non-traditional sourdough for the manufacturing of quinoa-wheat composite bread with increased biological value and improved sensory properties and acceptability.

The aim of this research was to assess the influence of three starter cultures, *Lactobacillus acidophilus* DSM 20079, *Lactobacillus plantarum* MR24, and *Lactobacillus brevis* R26, on the biochemical features as pH, total titratable acidity (TTA), volatile acidity, D/L-lactic acid, LAB count, enzyme activities (protease, amylase, phytase and cellulases) and antioxidant activity as well as TPC in fermented quinoa, and its effect on the quality and acceptability of quinoa-wheat composite bread. In addition to TPC, the antimicrobial and antioxidant activities of protein fractions isolated from fermented and unfermented quinoa were investigated.

## 2. Materials and Methods

### 2.1. Microorganisms

LAB strains: *L. acidophilus* DSM 20079, *L. plantarum* MR24 and *L. brevis* R26 were cultured in DeMan, Rogosa and Sharpe (MRS) broth (CM 0359, Oxoid Ltd., Basingstoke, Hampshire, UK) at 30 °C for *L. plantarum* MR24, and 37 °C for *L. brevis* R26 and *L. acidophilus* DSM 20079 for 24 h. *L. plantarum* MR24 and *L. brevis* R26 have been previously isolated from rye sourdough [27]. *L. acidophilus* DSM 20079 was purchased from Leibniz Institute DSMZ-German Collection of Microorganisms and Cell Cultures.

### 2.2. Fermentation of Quinoa Flour

Quinoa (*Chenopodium quinoa* Willd.) seeds were purchased from a local supermarket. Quinoa composition: 64.16 g of carbohydrates, 0 g of sugars, 6.7 g of fat, 0.7 g of saturated fatty acids, 14.12 g of crude protein, 0.12 g of salt, and 14.2 g of water; the country of origin was Bolivia. Quinoa seeds were milled (Vitek, An-Der, Austria) using a 500 μm size sieve. The obtained flour was sterilised in autoclave at 121 °C for 15 min to remove viable microorganisms. For fermentation, sterilised flour was mixed with sterile water to obtain a final moisture content of 50% (water activity was 0.932 ± 0.002). Fresh overnight LAB cultures (LAB count was in the range of 1.3–2.7 × 10^9^ CFU/mL) (0.2%) were used for inoculation on flour/water mixture. The obtained quinoa/water medium was fermented with a single LAB strain at 30 °C (using *L. plantarum* MR24) and 37 °C (using *L. brevis* R26 and *L. acidophilus* DSM 20079) for 72 h. Characteristics such as LAB count, TTA, pH, D/L-lactate content, volatile acidity, enzymatic activities, TPC and antioxidant activity were analysed in fermented quinoa. Additionally, TPC, antioxidant and antimicrobial activities were analysed in protein fractions isolated from fermented quinoa.

### 2.3. Determination of the Characteristics of Fermented Quinoa Flour

LAB counts in fermented quinoa flour were evaluated according to ISO 4833:2003 [28] with some modifications. MRS agar was used for LAB count evaluation. The Petri plates were incubated for 72 h at 30 °C (for *L. plantarum* MR24) and 37 °C (for *L. brevis* R26 and *L. acidophilus* DSM 20079) under an anaerobic atmosphere (Sigma Aldrich Co., St. Louis, MO, USA). The count of LAB in fermented quinoa flour was expressed as CFU/g.

The TTA of the fermented quinoa and quinoa-wheat composite breads was evaluated according to the standard techniques [29]. Samples (10 g) were homogenised with water (90 mL) in a porcelain mortar. The TTA was reported as millilitres of 1 M NaOH solution required to neutralise a sample to a pH of 8.5 [29].

For the determination of D/L-lactate, an enzymatic test K-DLATE 08/11 from Megazyme Ltd. (Wicklow, Ireland) was applied. Sample extract was prepared by mixing fermented quinoa sample (1 g) with water (80 mL), filtering through a Whatman’s No. 1 (Sigma Aldrich, St. Louis, MO, USA) and diluting with water up to 100 mL. The determination of D/L-lactate was carried out according to the manufacturer’s recommendations.

For the evaluation of volatile acidity, fermented quinoa sample (25 g) was transferred to a distillation flask with 2% sulphuric acid (7.5 mL) and water (50 mL). Distillation was carried out in a Behr S4 Distillation unit (Behr Labor-Technik GmbH, Düsseldorf, Germany) under a selected program (80% power, 540 s duration). The solution of 0.1 M NaOH was used for titration of the obtained distillate to a pH of 8.5. The volume of 0.1 M NaOH solution applied for the neutralisation was expressed as millilitres of 1 M NaOH solution needed for the neutralisation (to a pH of 8.5) of volatile acids present in 100 g of sample.

The antioxidant activity and TPC in fermented quinoa samples were expressed as equivalents of Trolox (TE) in mg per 100 g of quinoa and as equivalents of Gallic acid (GAE) in mg per 100 g of quinoa, respectively. For the evaluation of antioxidant activity and TPC, fermented and unfermented quinoa samples (0.5 g) were prepared in aqueous methanol (4 mL) (ratio 1:3) using a shaker for 15 h and filtered through the filter. Further analyses were carried out as described in paragraphs 2.5.6 and 2.5.7.

### 2.4. Determination of Enzymatic Activity in Fermented Quinoa Flour

Amylase activity was evaluated by measuring the intensity of starch-iodine solutions as described by Xiao et al. [30] with some modification described by Cizeikiene et al. [31]. One unit of amylase can catalyse the hydrolysis of 1 mg of soluble starch into dextrins in 1 min at 30 °C and pH 7.0. Fermented quinoa sample (0.1 g/mL) was homogenised with Na_2_HPO_4_ buffer (0.1 M, pH 7.0), filtered through Whatman’s No. 1. Sample extract (1 mL) and 1 mg/mL starch (Sigma Aldrich) solution (1 mL) was mixed. As a control sample, Na_2_HPO_4_ buffer (1 mL) was mixed with 1 mg/mL starch solution (1 mL). For blank, Na_2_HPO_4_ buffer (1 mL) was mixed with sample extract (1 mL). The reactions were performed at 30 °C for 30 min, and stopped by adding 1 M HCl solution (0.5 mL), after which 1 M iodine solution (2.5 mL) (5 M KI and 5 M I_2_) was added. The absorbance was measured at 580 nm.

Phytase activity was determined by combining the methods of Quan et al. [32] and Olstorpe et al. [33]. One unit of phytase is the quantity of enzyme liberating 1 μmol phosphorus from potassium phytate (Sigma Aldrich) per min at 30 °C and pH 5.5. Fermented quinoa sample (0.1 g/mL) was prepared in NaCOOCH_3_ buffer (0.2 M, pH 5.5). The reaction mix consisted of NaCOOCH_3_ buffer (0.8 mL) supplemented with 3 mM potassium phytate and sample extract (0.2 mL). For the blank, NaCOOCH_3_ buffer (0.8 mL) was mixed with sample extract (0.2 mL). The reaction was performed at 30 °C for 30 min. The reactions were stopped by adding 10% trichloroacetic acid (1 mL). Afterwards, the reaction mixture (0.2 mL) was mixed with a colour reagent (1.6 mL) 10 mM (NH_4_)_6_Mo_7_O_24_∙4H_2_O:2.5 M H_2_SO_4_:acetone (ratio 1:1:2) and the absorbance was measured at 355 nm after 20 min.

Cellulase activity was determined by assessing the total content of reducing sugars. One unit of cellulases is characterised as the quantity of enzyme releasing 1 μmol of glucose from cellulose filter paper per min at 30 °C and pH 4.8. Fermented quinoa sample (0.1 g/mL) was prepared in citric buffer (0.05 M, pH 4.8). Sample extract was obtained after filtration and used for the determination of cellulase activity. The reaction mix was prepared from sample extract (0.1 mL), cellulose filter paper (1.0–6.0 cm^2^) and citric buffer (0.9 mL). For the blank, citric buffer (0.9 mL) was mixed with sample extract (0.1 mL). The reaction was performed at 30 °C for 30 min. Afterwards, the reaction mixture (1 mL) was mixed with a reagent of 3.5-dinitrosalicylic acid (1 mL) and boiled for 5 min in water bath, then cooled, and the absorbance was measured at 540 nm.

Protease activity was measured using casein as a substrate [34]. One unit of protease is the content of tyrosine (μmol) liberated from casein (Sigma Aldrich) for one min at 37 °C and pH 7.5. Fermented quinoa sample (0.1 g/mL) was homogenised in NaCOOCH_3_ buffer (10 mM, pH 7.5) supplemented with 5 mM CaCl_2_ and filtered for the determination of protease activity. Sample extract (1 mL) and 0.65% casein solution (5 mL, pH 7.5) was mixed and the reaction was performed at 37 °C for 10 min. Afterwards, the enzymatic reaction was inactivated by adding 110 mmol/L trichloroacetic acid solution (5 mL). As a blank sample, sample extract (1 mL) was added immediately after the trichloroacetic acid solution. Then the test tubes with solutions were heated at 37 °C for 30 min afterwards, a 0.45 µm polyethersulfone syringe filters (Sigma Aldrich) were used for the filtration of solutions. Then, the obtained filtrate (2 mL) was mixed with 0.5 M Na_2_CO_3_ (5 mL) and 0.5 M Folin & Ciocalteu’s phenol reagent (Sigma Aldrich) (1 mL), maintained for 30 min and filtered. The absorbance of filtered solutions was measured at 660 nm.

### 2.5. Isolation of Protein Fractions from Fermented Quinoa, Hydrolysis and Bioactive Compounds Determination

#### 2.5.1. Defatting of Quinoa Samples for Isolation of Protein Fractions

For protein fractions isolation, fermented quinoa flour was dried in a drying oven (Mechanical convection, Thermo Fisher Scientific) at 30 °C overnight. Unfermented flour was used as a control sample for further experiments. Defatting of fermented and unfermented quinoa flour was carried out according to Elsohaimy et al. [14] with slight changes. A triple chloroform:methanol (ratio 2:1) extraction was carried out. Quinoa sample was mixed with chloroform:methanol solution (ratio 1:5, *w*/*v*) for 2 h at 23 °C using a shaker (IKA KS 130). After the final extraction, the chloroform:methanol solution was removed and the rest was evaporated in a fume hood at 23 °C for 16 h. Defatted samples were used to isolate protein fractions according to their solubility in different solvents: water, salt solution and ethanol.

#### 2.5.2. Water-Soluble Protein Fraction Isolation

The isolation of water-soluble protein fraction was carried out according to Elsohaimy et al. [14] with slight modifications. Defatted quinoa flour was mixed with distilled water (1:20, *w*/*v*). The pH of sample was adjusted to 10 using 0.1 M NaOH. The obtained mixture was stirred for 1.5 h and pH was maintained at 10 to obtain the maximum level of protein solubilisation. Then, the mixture was centrifuged (6000 rpm, 15 min, 4 °C) and supernatant was collected. Next, 1 M hydrochloric acid was added to the supernatant using continuous stirring until the pH of the solution reached 4.5. Precipitated proteins were collected using centrifugation (8000 rpm, 30 min, 4 °C), frozen at −18 °C, freeze-dried (ZIRBUS Sublimator 3 × 4 × 5, Bad Grund, Germany) and stored at −18 °C.

#### 2.5.3. Extraction of Salt-Soluble Protein Fraction

The protein fraction soluble in 0.8 M NaCl was isolated as described by Hadnađev et al. [35] with some modifications. Defatted flour was mixed with NaCl solution (0.8 M, pH 7.0) at 1:10 (*w*/*v*), stirred for 2 h at 23 °C and centrifuged (6000 rpm, 10 min, 4 °C) using 10 kDa membranes (Amicon Ultra, Merck Millipore). The precipitates were washed twice with distilled water and centrifuged using 10 kDa membranes. Obtained protein pellets were frozen at −18 °C, freeze-dried (ZIRBUS Sublimator 3 × 4 × 5, Bad Grund, Germany) and stored at −18 °C.

#### 2.5.4. Extraction of Ethanol-Soluble Protein Fraction

Defatted flour was mixed with 70% ethanol (ratio 1:5, *w*/*v*), stirred for 5 h at 23 °C, and filtered through Whatman’s filter paper according to Siddeeg et al. [19]. Ethanol was removed using a rotary evaporator (RV 10 Basic IKA, Staufen, Germany) (170 rpm/min, 40 °C). The solution obtained after evaporation was frozen at −18 °C, freeze-dried (ZIRBUS Sublimator 3 × 4 × 5, Bad Grund, Germany) and stored at −18 °C.

#### 2.5.5. Enzymatic Hydrolysis of Protein with Pepsin

Protein fractions: (i) soluble in water, (ii) soluble in ethanol, and (iii) soluble in 0.8 M NaCl were hydrolysed with a pepsin according to Siddeeg et al. [19]. The freeze-dried fractions were dissolved in water at pH 2 with 1% protein. The pepsin (Sigma-Aldrich, St. Louis, MO, JAV) solution (10 mg/mL) was dissolved in water at pH 2. The prepared pepsin solution was mixed with protein solution at a ratio of 1:100 (*v*/*v*). Hydrolysis was carried out at 37 °C for 3 h. Afterwords, pepsin was inactivated at 95 °C for 15 min in a water bath, and then cooled; the pH was adjusted to 7. Protein fractions hydrolysed with pepsin were used for ABTS radical scavenging assay, antimicrobial analysis and the TPC assay.

#### 2.5.6. ABTS Radical Scavenging Assay

The radical scavenging activity of protein fractions and enzymatically hydrolysed protein fractions was evaluated by ABTS radical cation decolourisation assay according to Rajurkar and Hande [36]. ABTS^+^ cation radicals were produced by the reaction of 2.45 mM potassium persulphate and 7 mM ABTS in water at a ratio of 1:1 and kept in the dark for 16 h before use at room temperature. The obtained ABTS^+^ solution was diluted with methanol to reach an absorbance of 0.700 at 734 nm spectrophotometrically. ABTS^+^ solution (3.995 mL) was mixed with a protein fraction (5 µL) (10 mg protein per ml distilled water) and kept in the dark room. After 30 min, the absorbance was measured at 734 nm. Trolox (0.05–1.00 mg/mL) was used as standard to obtain a calibration curve. The antioxidant activity in protein fractions was expressed as equivalents of Trolox (TE) in mg per 100 g of protein.

#### 2.5.7. Determination of TPC in Protein Fractions

TPC in protein fractions and enzymatically hydrolysed protein fractions were determined using the modified Folin-Ciocalteu’s method [37]. Protein samples (100 µL) (10 mg protein per ml distilled water) were mixed with 3.3% sodium carbonate solution (3000 µL) and Folin-Ciocalteu’s phenol reagent (100 µL). The mixture was kept at room temperature for 30 min and the absorbance was measured at 760 nm. Gallic acid (0.01–1.00 mg/mL) was used as a standard to obtain the calibration curve. TPC in protein fractions was expressed as equivalents of Gallic acid (GAE) in mg per 100 g of protein.

#### 2.5.8. Determination of Antimicrobial Activity of Protein Fractions

The antimicrobial activity of protein fractions and enzymatically hydrolysed protein fractions against food spoilage bacteria were evaluated by the agar diffusion method, as described by Cizeikiene et al. [38] with slight modifications. The bacteria *Staphylococcus aureus*, *Bacillus subtilis*, *Bacillus cereus*, *Escherichia coli*, *Salmonella typhimurium* were obtained from the Department of Food Science and Technology (Kaunas University of Technology). Bacteria were grown for 24 h on Plate Count Agar (Biolife, Monza, Italy) in an incubator at 37 °C. Bacteria were collected from agar slants after growing, to make inocula containing ~10^8^ cells/mL of bacteria using McFarland 0.5 standard. Into Petri dishes, 133 µL of the indicator strain suspension was poured and overlaid with 20 mL of soft agar medium (Plate Count Agar). Seventy µl of protein fractions or enzymatically hydrolysed protein fractions (10 mg per ml distilled water) were added to each well (diameter 6 mm) that had been cut in the agar plates and kept for 24 h at 30 °C. The antimicrobial activity of protein fractions were evaluated against bacteria by measuring the growth inhibition zone in millimetres. Antimicrobial activity (the inhibition of bacteria growth around the well) was evaluated using the following scale: ‘0′—no clear zone around the well—no inhibition; ‘+/−‘—up to 1 mm clear zone; ‘+’—1–3 mm clear zone; ‘++’—3–5 mm clear zone; ‘+++’—>5 mm clear zone.

### 2.6. The Preparation of Quinoa-Wheat Composite Bread

Ingredients for quinoa-wheat composite bread were purchased from a supermarket. Control wheat bread (CB) was produced using 1 kg of wheat flour with a gluten content of 25–27% (Malsena Plius Ltd., Vievis, Lithuania), 15 g of iodised sea salt (Droga, Portorose, Slovenia), 100 g of sugar (Nordic Sugar Kedainiai Ltd., Kedainiai, Lithuania), 25 g of fresh compressed yeast for baking (Lallemand Baltic Ltd., Panevėžys, Lithuania), 30 g of sunflower oil (Anira Ltd., Kaunas, Lithuania), and water (for all dough preparation water content was re-calculated to obtain 47% dough moisture content). Wheat bread with fermented quinoa was made by replacing 5 and 10% (by weight) of wheat flour with wet quinoa flour previously fermented with *L. brevis* (5% FQB and 10% FQB respectively). Quinoa-wheat composite bread supplemented with unfermented quinoa flour and seeds were made by substituting 5% (by weight) of wheat flour with quinoa flour and quinoa seed (5% QFlB and 5% QSB, respectively). The dough was kneaded for 2 min in a stirrer (ELBA 7 W NEW, Fiamma Sdn Bhd, Kuala Lumpur, Malaysia) at the lowest speed and 8 min using the highest speed. The obtained dough was fermented at 30 °C (relative humidity (RH) of 85%) for 30 min, divided into portions (80 g) and rounded. Proofing was carried out at 35 °C (RH of 85%) for 45 min. Breads were baked at 200 °C for 20 min in a baking oven (MIWE Michael Wenz GmbH, Arnstein, Germany).

Different types of quinoa-wheat composite bread were made: control wheat bread without any quinoa (CB); wheat bread with 5% of unfermented quinoa flour and 5% of quinoa seed (5% QFlB and 5% QSB, respectively); and wheat bread prepared with 5 and 10% of quinoa flour previously fermented with *L. brevis* (5% FQB and 10% FQB respectively). Measurements of the quality characteristics of the quinoa-wheat composite bread were taken 18 h after baking.

### 2.7. Evaluation of Quinoa-Wheat Composite Bread Characteristics

Bread specific volume was determined according to the AACC 10–05.01 [29]. Millet grit (Skanėja, Vilnius, Lithuania) was used instead of rapeseed, as described by Cizeikiene et al. [31]. Porosity of bread crumb was measured with a Zhuravlev device (Biomer Ltd., Krasnoobsk, Russia). The TTA of the wheat bread was evaluated according to the standard techniques [29] as described above (paragraph 2.3). Sensory properties of wheat breads were evaluated using a 7-point rating scale by 15 panellists (7 males and 8 females aged between 22 and 35) 18 h after baking. The lowest intensity of the attribute (overall odour, overall flavour, moistness, crumbliness, foreign flavour, and foreign odour) corresponded to a value of 1 and the highest intensity corresponded to the value of 7. Wheat bread samples were evaluated for overall acceptability using a 7-point hedonic scale, where point 7 was ‘like extremely’ and point 1 was ‘extremely dislike’. Blinded samples without crusts were cut in slices (thickness about 1 cm).

### 2.8. Statistical Analysis

Three independent replications were carried out for all experiments. The software package STATISTICA 10.0 (StatSoft Corp., Krakow, Poland) was applied to evaluate if variables varied between the control and analysed sample by applying the Duncan post-hoc test (*p* < 0.05).

## 3. Results and Discussion

### 3.1. Characteristics of Fermented Quinoa

#### 3.1.1. LAB Adaptation and the Acidity Parameters of Fermented Quinoa

The main parameters observed during quinoa fermentation were counts of LAB and acidity parameters such as TTA, pH, lactic and acetic acid contents, antioxidant activity and TPC (Table 1).

The results confirmed that *L. plantarum*, *L. brevis*, and *L. acidophilus* were well adapted in the quinoa flour environment. The maximum count of LAB was in quinoa fermented using *L. acidophilus* as a starter ((3.2 ± 0.1) × 10^10^ CFU/g) used as a single strain. Furthermore, high numbers of LAB were in fermented quinoa with *L. plantarum* ((3.9 ± 0.3) × 10^9^ CFU/g) and *L. brevis* ((8.05 ± 0.1) × 10^9^ CFU/g), confirming the suitability of quinoa flour for LAB multiplication and non-traditional sourdough production. LAB have complex nutritional requirements for carbohydrates, peptides, amino acids, fatty acids, vitamins and salts [39], as results show that quinoa is a suitable environment for LAB growth. The count of LAB used for quinoa flour fermentation were similar to count of traditional sourdough. During traditional sourdough (rye or wheat) fermentation, the counts of specially applied LAB were found to be 10^8^–10^9^ CFU/g and the number of LAB depend on flour type, temperature and starter cultures [39]. According to Lönner and Preve-Åkesson [40] the regular sourdough should contain >5·10^8^ CFU/g metabolically active LAB and pH should be <4.5. In quinoa sourdough made with *L. plantarum* and *L. acidophilus* the pH were 4.3 and 4.4 respectively, and TTA were 9.2 and 8.1 mL, respectively. This confirms the suitability of those strains for non-traditional sourdough production. The typical TTA of wheat sourdough and whole-grain sourdough has been reported to be 8–13 and 16–22 mL, respectively [39,41]. Wheat sourdough with lower TTA (3.47–4.5 mL) and higher pH (4.77–5.17 mL) was reported by Nisa et al. [42]. Similar results were obtained in quinoa fermented with *L. brevis*.

The maximum total lactic acid content was measured in quinoa sourdough made with *L. acidophilus*, while in sourdough with *L. plantarum* and *L. brevis*, the total lactic acid content was 2.3 and 3.5 times less, respectively. Volatile acidity represents the content of acetic acid, whereas there were no significant differences found in the volatile acidity between fermented quinoa and the tested LAB strains. LAB produce lactic and acetic acids as the major products during the fermentation of carbohydrates, resulting in a decreased sugar content and decreased pH in sourdough. Species belonging to *Lactobacillus* genus were intended for L(+) lactic acid production [43]; however, in this study, used LAB produced a combination of lactic acid isomers (L(+) and D(−)), and the major isomer was L(+) lactic acid in fermented quinoa. According to Tanyıldızı et al. [44] microbial production of lactic acid isomers forms either as a mixture in different proportions or separately, and depends on the substrate, starter cultures and growth conditions.

#### 3.1.2. Enzymatic Activities in Fermented Quinoa

Starch and non-starch carbohydrate hydrolysing enzymes are often applied in bread production technological processes to modify the dough rheology properties, improve the quality of bread, and retard staling [45,46]. α-amylase is the most commonly used enzyme, but cellulases and proteases are also used in practice. Phytase producing LAB is also favoured in wheat bread production, particularly in the whole-grain cereal goods manufacturing process because of their capacity to increase the nutritional rate [26,27]. The ability of LAB to produce phytase and therefore to release phosphate and minerals from the phytate complex in cereal medium is a desirable property of starter strains used for cereal fermentation. *L. plantarum* MR24, *L. brevis* R26 and *L. acidophilus* DSM 20079 cause the phytase activity in quinoa flour medium (Table 1). However, *L. acidophilus* DSM 20079 showed significantly higher phytase activity in whole-grain wheat medium [31] compared to quinoa. Even the major studies have described LAB strains possessing intracellular phytase activities; it is implausible that intracellular phytase participates in cereal phytic acid degradation [47]. Therefore, extracellular phytase producing LAB is favourable for cereal product fermentation. Since enzyme activity is influenced by fermentation conditions, evaluation methods, and defined units, it is difficult to compare obtained results of different studies.

Cellulase activities (Table 1) in quinoa fermented with *L. plantarum* and *L. brevis* were 28.2 and 37.1% higher than quinoa fermented with *L. acidophilus*, respectively. Amylase activities in quinoa fermented with *L. plantarum* and *L. acidophilus* were 75.4 and 95.5% lower, respectively, than in quinoa fermented with *L. brevis*. Low protease activities were found in quinoa fermented with *L. plantarum* and *L. brevis*, whereas in quinoa fermented with *L. acidophilus*, the protease activity was not found. Other studies showed the proteolytic activity of strains belonging to *L. acidophilus* species in a varied range [48,49,50]. Microbial proteases are important in the hydrolysis of proteins resulting smaller peptides and in the following degradation into free amino acids [51], it forms intermediate compounds during the production of aromatic products [52]. In bread making, proteases influence the physical properties of dough and quality of bread. Dough becomes more extensible and develops at a faster rate. An excessive amount of proteases weakens the dough and lowers the ability of gas-retaining, therefore it must be controlled. The results confirmed that the desired properties of sourdough can be obtained depending on the LAB strain used for quinoa flour fermentation.

Considering that quinoa fermented with *L. brevis* showed the highest amylase, protease, cellulase and phytase activities, this product was selected for the production of quinoa-wheat composite bread.

### 3.2. Characteristics of Protein Fractions from Fermented Quinoa

#### 3.2.1. Antioxidant Activity and TPC in Protein Fractions from Quinoa

The antioxidant activity of plant peptides is associated with the composition of amino acids, its chain length, conformation and sequence, hydrolysis degree [18,53]. During this study, we assessed the antioxidant activity (Table 2) of different protein fractions from quinoa flour obtained after: (i) fermentation with LAB and (ii) enzymatic hydrolysis with pepsin from the protein fractions.

The fermentation of quinoa with LAB influenced the antioxidant activity of protein fractions from quinoa. The highest antioxidant activities were observed in protein fractions soluble in 70% ethanol. Moreover, the highest TPC was observed in ethanol fraction revealing that phenolic compounds bound to proteins were precipitated in this fraction. The antioxidant activity of protein fraction soluble in 70% ethanol could be highly increased by protein conjugation with phenolic compounds [54]. Antioxidant activity of phenolic–protein conjugates is effected by the amount of phenols covalently bounded to proteins and it is strongly related to the sort of phenolic compound attached [54]. Nwachukwu and Aluko [55] noticed that differences in the peptide conformation which could also have contributed to the evaluation of antioxidant capacities. Ma et al. [56] revealed that conformation or even the spatial structure of peptide chains had a high influence on the antioxidant activity. Furthermore, there might be some synergistic antioxidant activities between phenolic compounds and proteins or peptides.

The antioxidant activity of ethanol soluble protein faction obtained from quinoa fermented with *L. acidophilus* was the highest compared with same fraction from unfermented quinoa and quinoa fermented with *L. brevis* and *L. plantarum*. Quinoa fermentation with *L. brevis* reduced the antioxidant activities of water soluble and salt soluble protein fractions. Fermentation with LAB may influence TPC and antioxidant activity, mainly due to the breakdown of the cell wall of grains; the liberation of attached phenols may influence the increasing antioxidant activities by the action of enzymes [57].

Hydrolysis with pepsin strongly reduced the antioxidant activity of protein fractions in all cases, which confirms that the antioxidant effect was due to proteins and that hydrolysis by pepsin altered the structure of the primary proteins. In particular, in water soluble and salt soluble protein fractions, the antioxidant activity was totally lost after treatment with pepsin. In the contrast, it was reported that the DPPH radical scavenging activity of the quinoa protein hydrolysate (produced with alcalase and pepsin) was higher compared to the native parental proteins [16,18]. Zhidong et al. [58] reported that the increasing hydrolysis duration and enzyme ratio of the hydrolysis resulted in the antioxidant properties increasing following a decline; therefore, hydrolysis time and enzyme/substrate ratio should be under control. Different results were reported by Rival et al. [59], which show that the antioxidant activity of the bovine casein hydrolysates (obtained using different proteases) was lower than that of native proteins.

The fermentation of quinoa with LAB influenced TPC in all quinoa protein fractions. *L. plantarum* and *L. acidophilus* fermentation increased TPC in water-soluble protein fraction, whereas *L. brevis* fermentation reduced TPC. A high increase of TPC was found in the ethanol-soluble protein faction and salt-soluble protein fraction obtained from quinoa fermented with *L. acidophilus* as well as the antioxidant activity. Hydrolysis with pepsin strongly reduced the TPC in protein fractions except in the water-soluble protein fraction obtained from unfermented quinoa and quinoa fermented with *L. brevis*. Reduced TPC in protein fractions after hydrolysis with pepsin may be explained as the liberated peptides may bind to phenolic compounds and form new peptide-phenol complexes. Some studies showed that antioxidant activity could be reduced by the covalent and non-covalent interactions between phenols and proteins [60,61,62].

A strong positive correlation was found between the antioxidant activity of non-hydrolysed protein fractions of quinoa and TPC (*R*^2^ = 0.985), and between antioxidant activity of protein fractions obtained after hydrolysis with pepsin and TPC (*R*^2^ = 0.871). TPC in quinoa strongly depend on the variety and processing of quinoa seed [63].

#### 3.2.2. Antimicrobial Activity of Protein Fractions from Quinoa

**Figure 1:**
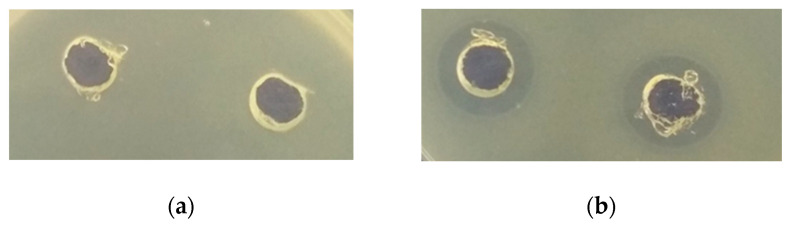
Antimicrobial effect of water-soluble protein fractions from quinoa fermented with *L. plantarum* MR24 against *E. coli* growth in agar plates. The clear zone of inhibition around the “well” indicates inhibition growth of *E. coli*: (**a**)—non-hydrolysed protein fraction; (**b**)—protein fraction hydrolysed with pepsin.

The antimicrobial activity of protein fractions from quinoa depended on the LAB strain used and spoilage bacteria. Antimicrobial activity of protein fractions was higher after hydrolysis with pepsin (Table 3). In many cases, fermentation with LAB increased the antimicrobial activity of protein fractions from quinoa. The highest antimicrobial activity showed ethanol-soluble protein fractions after the hydrolysis with pepsin. The antimicrobial effect of water-soluble protein fractions from quinoa fermented with *L. plantarum* MR24 against *E. coli* growth is shown in Figure 1.

Non-hydrolysed water-soluble and salt-soluble protein fractions did not show antimicrobial activity against the tested microorganisms with the exception of the non-hydrolysed salt-soluble fraction from quinoa fermented with *L. acidophilus* (weakly inhibited the growth of *B. subtilis*) and non-hydrolysed salt-soluble fraction from unfermented quinoa (weakly inhibited the growth of *B. cereus*). Unfermented and non-hydrolysed protein fractions from quinoa did not show antimicrobial activity except for salt-soluble protein fractions (weakly inhibited the growth of *B. cereus*) and ethanol-soluble protein fractions (weakly inhibited the growth of *B. subtilis*). Other researchers evaluated the antimicrobial activities of quinoa extracts. Park at al. [64] determined the weak antimicrobial activity of the quinoa seed extracts against *Staphylococcus aureus*, *Bacillus cereus*, *Escherichia coli*, *Salmonella typhimurium*, and *Campylobacter jejuni*, whereas no activity was determined against *Listeria monocytogenes*. Miranda et al. [65], found the strong antimicrobial activity of quinoa seed against *E. coli* (8.29–14.79 mm) and *S. aureus* (8.53–15.03 mm).

### 3.3. Characteristics and Sensory Evaluation of Breads Made with Quinoa Additives

Quinoa fermented with *L. brevis* showed the highest amylase, protease, cellulase and phytase activities; therefore, it was selected for quinoa-wheat composite bread production. Among the various characteristics of bread, specific volume and crumb porosity are two important visual features that strongly influence the consumer’s choice. The characteristics of breads made with fermented and unfermented quinoa additives are shown in Table 4. The highest crumb porosity was in bread made with 5% of unfermented quinoa flour and with 5% of unground quinoa seed. Fermented quinoa additives slightly reduced the porosity of bread. However, these differences were not statistically significant. Quinoa additives increased bread specific volume in the range from 3–11%. The maximum specific volume was of bread made with 5% of unfermented quinoa flour and bread made with 10% of fermented quinoa with *L. brevis*. Low amounts of quinoa additive did not show a negative impact on bread quality. According to Wyrwisz [66], dietary fiber has a negative influence on bread quality characteristics, such as a decreasing volume by lowering the gas retention.

Another important characteristic for consumers is the acidity of bread. The additives of quinoa fermented with *L. brevis* did not increase the TTA of breads compared with the control bread (bread made without quinoa additive) and with bread made with unfermented quinoa flour. Whereas the additives of quinoa whole seed reduced the TTA by 18%. The results indicated that the addition of 5 and 10% of quinoa fermented with *L. brevis* would not affect the TTA of wheat bread, while 10% of fermented quinoa resulted in a higher specific volume. The addition of sourdough usually increased the TTA of dough and bread [27,37,39]; the increase depended on the amount of sourdough, starter culture used, flour type used for sourdough, and the temperature used for sourdough fermentation. Fermented quinoa with a low initial pH and TTA (respectively 4.6 and 5.5) had no significant influence on bread acidity.

**Figure 2:**
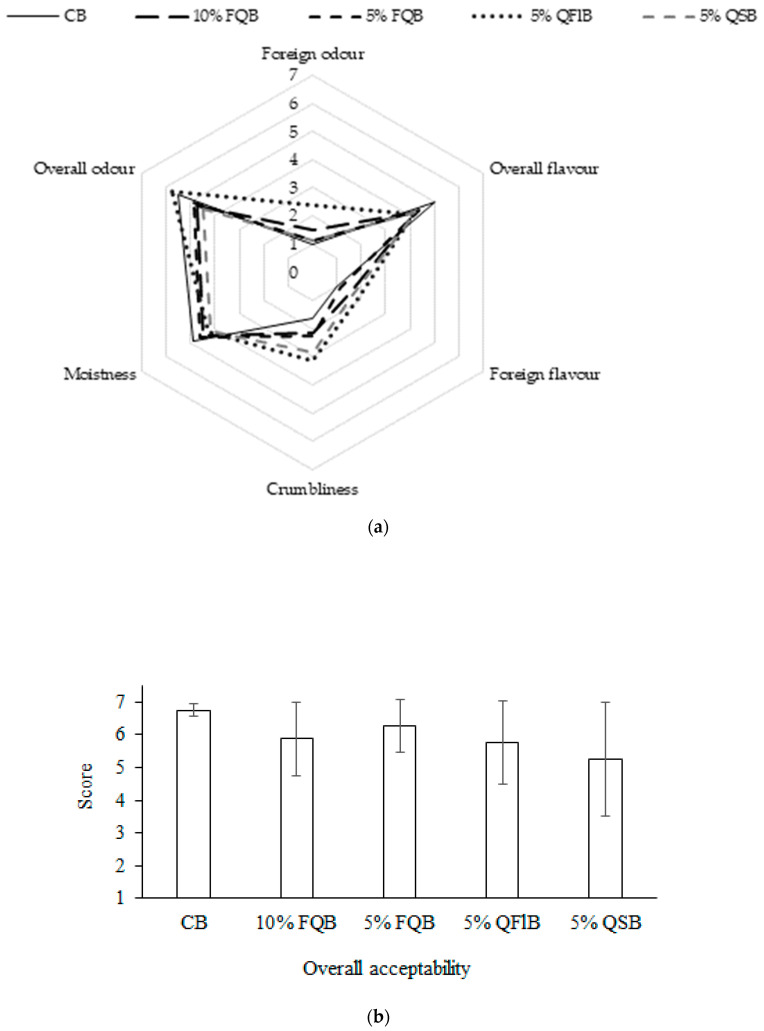
Quinoa additives effect on sensory properties (**a**) and overall acceptability (**b**) of quinoa-wheat composite bread.

Sensory characteristics are significant reasons influencing consumer acceptance. Furthermore, sensory characteristics are a key to success in marketing. The evaluation of sensory properties (overall odour, foreign odour, overall flavour, foreign flavour, crumbliness and moistness) of wheat breads made with quinoa additives is shown in Figure 2a. The panellists detected that all kind additives of quinoa reduced moistness, overall flavour and increased foreign odour, and the foreign flavour crumbliness of breads compared with a control bread. The highest crumbliness was of breads made with unfermented quinoa flour and seeds. The panellists noticed that the highest foreign odour was shown for the bread with 5% of unfermented quinoa flour and unground quinoa seed. Fermented quinoa additives increased the overall acceptability of bread compared with the bread made with the same amount of unfermented seed flours (Figure 2b).

## 4. Conclusions

In conclusion, the starter cultures *L. acidophilus* DSM 20079, *L. plantarum* MR24, and *L. brevis* R26 could be applied for quinoa fermentation. Biochemical properties like TTA, pH, volatile acidity, D/L-lactic acid, LAB count, enzyme activities (protease, amylase, phytase and cellulase), TPC and the antioxidant activity of fermented quinoa depended on the LAB strain used as a starter for fermentation. Additives of fermented quinoa with *L. brevis* could be recommended for quinoa-wheat composite bread production with higher TPC, antioxidant activity and higher overall acceptability of bread compared with unfermented seed additives. The protein fractions from fermented quinoa exhibited a high antioxidant activity to be integrated in food products. The antioxidant activity of protein factions obtained after quinoa fermentation with *L. acidophilus* were the highest compared with the other LAB or unfermented seed used. *L. brevis* produced the highest phytase activity in quinoa medium therefore, quinoa fermented with these LAB could be recommended for bread production to make nutritionally fortified bread with good sensorial properties and acceptability. The results showed the ability of LAB to increase TPC and antioxidant activity through the proteolysis of LAB produced enzymes in quinoa flour after fermentation. The application of fermented quinoa flour with *L. brevis* might be considered relevant for novel applications as a functional dietary supplement.

## Figures and Tables

**Table 1:** Characteristics of quinoa fermented with different LAB strains for 72 h.

	*Unfermented quinoa*	*L. plantarum* MR24	*L. brevis* R26	*L. acidophilus* DSM 20079
LAB count, CFU/g	-	(3.9 ± 0.3) × 10^9^ a	(8.05 ± 0.1) × 10^9^ b	(3.2 ± 0.1) × 10^10^ c
*Acidity*				
pH	6.6 ± 0.1 c	4.3 ± 0.05 a	4.6 ± 0.05 b	4.4 ± 0.04 a
TTA, mL of 1 M NaOH	2.6 ± 0.02 ca	9.2 ± 0.02 d	5.5 ± 0.03 b	8.1 ± 0.05 c
Volatile acidity, mL of 1 M NaOH	0.02 ± 0.01 a	3.00 ± 0.04 b	3.00 ± 0.10 b	2.88 ± 0.09 b
*Lactic acid content*				
L-lactic acid, g/kg	2.3 ± 0.03 a	13.0 ± 0.24 c	9.2 ± 0.21 b	42.5 ± 0.26 d
D-lactic acid, g/kg	0.5 ± 0.01 a	6.7 ± 0.15 d	3.4 ± 0.07 c	2.1 ± 0.04 b
*Enzymatic activities*				
Cellulase activity, CU/g	0	0.577 ± 0.022 b	0.617 ± 0.065 b	0.450 ± 0.071 a
Amylase activity, AU/g	0.259 ± 0.05 c	0.082 ± 0.006 b	0.333 ± 0.008 d	0.015 ± 0.001 a
Phytase activity, PhU/g	0	0.125 ± 0.004 b	0.142 ± 0.003 c	0.100 ± 0 a
Protease activity, PU/g	0	0.038 ± 0.008 a	0.042 ± 0.003 a	0
*TPC and antioxidant activity*				
TPC, GAE mg/100 g	32.3 ± 0.2 a	39.2 ± 0.5 b	61.4 ± 3.0 c	39.1 ± 1.4 b
Antioxidant activity, mg TE/100 g	37.6 ± 2.8 b	10.2 ± 0.6 a	53.4 ± 3.7 c	29.0 ± 3.2 b

The values in a row having a different characters (a–c) are significantly different (*p* < 0.05).

**Table 2:** Antioxidant activity (mg TE/100 g protein) and total phenolic content (GAE mg/100 g protein) in protein fractions from quinoa obtained after: (i) fermentation with LAB and (ii) enzymatic hydrolysis with pepsin.

LAB Used for Quinoa Fermentation	Antioxidant Activity		Total Phenolic Content		
	Non-Hydrolysed	After Hydrolysis with Pepsin		Non-Hydrolysed	After Hydrolysis with Pepsin
*Water-soluble protein fraction*					
Unfermented flour	34.8 ± 5.3 b	0		0.47 ± 0.02 a	1.92 ± 0.03 b
*L. plantarum*	64.1 ± 6.7 d	0		0.80 ± 0.02 b	0.33 ± 0.07 a
*L. brevis*	22.1 ± 4.1 a	0		0.27 ± 0.03 a	0.45 ± 0.04 a
*L. acidophilus*	52.2 ± 12.5 c	0		0.86 ± 0.02 b	0.58 ± 0.11 a
*Protein fraction soluble in 0.8 M NaCl*					
Unfermented flour	27.5 ± 6.4 a	0		0.88 ± 0.04 a	0.66 ± 0.03 a
*L. plantarum*	21.5 ± 3.6 a	0		0.90 ± 0.02 a	0.66 ± 0.02 a
*L. brevis*	26.1 ± 4.5 a	0		0.98 ± 0.01 a	0.65 ± 0.02 a
*L. acidophilus*	35.3 ± 6.5 b	0		1.37 ± 0.04 b	0.64 ± 0.01 a
*Protein fraction soluble in 70% ethanol*					
Unfermented flour	1109 ± 141 c	139.5 ± 21.6 d		50.1 ± 0.45 b	18.5 ± 0.38 d
*L. plantarum*	69.3 ± 16.5 a	9.1 ± 1.2 a		4.63 ± 0.21 a	3.43 ± 0.07 a
*L. brevis*	881.6 ± 95.8 b	24.8 ± 9.7 b		68.0 ± 4.16 b	11.2 ± 0.02 c
*L. acidophilus*	3261 ± 169 d	76.2 ± 14.3 c		186.1 ± 9.8 c	10.5 ± 0.03 b

The values in a column with different characters (a–d) are significantly different (*p* < 0.05).

**Table 3:** Influence of fermentation with LAB and enzymatic hydrolysis with pepsin on antimicrobial properties of protein fractions from quinoa.

Food Spoilage Bacteria	LAB Strain Used for Quinoa Flour Fermentation						Unfermented Flour	
	*L. plantarum*		*L. brevis*		*L. acidophilus*			
	Non-Hyd.	After Hyd. with Pepsin	Non-Hyd.	After Hyd. with Pepsin	Non-Hyd.	After Hyd. with Pepsin	Non-Hyd.	After Hyd. with Pepsin
	*Water-soluble protein fraction*							
*E. coli*	-	++	-	+/−	-	+/−	-	+
*St. aureus*	-	+	-	-	-	+/-	-	-
*S. typhimurium*	-	+	-	+/−	-	+/−	-	+
*B. subtilis*	-	++	-	+	-	+	-	++
*B. cereus*	-	+	-	-	-	+/−	-	+
	*Protein fractions soluble in 0.8 M NaCl*							
*E. coli*	-	-	-	+/−	-	+/−	-	-
*St. aureus*	-	+/−	-	-	-	-	-	-
*S. typhimurium*	-	+/−	-	+/−	-	+/−	-	-
*B. subtilis*	-	+/−	-	+	+/−	+	-	+
*B. cereus*	-	-	-	-	-	+/−	+/−	+/−
	*Protein fractions soluble in 70% ethanol*							
*E. coli*	-	++	-	+	+	++	-	+/−
*St. aureus*	+/−	++	+/−	+	+	++	-	+
*S. typhimurium*	++	++	+	++	+	++	-	+
*B. subtilis*	+	+++	+	++	+	++	+/−	+
*B. cereus*	+	++	+	++	+	++	-	+/−

Evaluation of antimicrobial activities: (-) no clear zone around the well (no inhibition); (+/−) up to 1 mm clear zone; (+) 1–3 mm clear zone; (++) 3–5 mm clear zone; (+++) >5 mm clear zone; hyd.—hydrolysis.

**Table 4:** Characteristics of quinoa-wheat composite breads.

Bread Samples	CB	10% FQB	5% FQB	5% QFlB	5% QSB
Porosity, %	78.1 ± 1.1 a	77.7 ± 0.9 a	77.3 ± 1.3 a	80.8 ± 0 a	80.5 ± 0.9 a
Specific volume, cm^3^/g	3.09 ± 0.16 a	3.32 ± 0.22 b	3.18 ± 0.11 ab	3.42 ± 0.17 b	3.27 ± 0.15 b
TTA, mL of 1 M NaOH	2.2 ± 0.02 b	2.2 ± 0.01 b	2.2 ± 0.01 b	2.0 ± 0.01 ab	1.8 ± 0 ac

Values in a row with a same letter are not significantly different (*p* ≤ 0.05). CB: control bread (without quinoa); 10% FQB: bread prepared with 10% of quinoa flour fermented with *L. brevis*; 5% FQB: bread prepared with 5% of quinoa fermented flour with *L. brevis*; 5% QFlB: bread with 5% of unfermented quinoa flour; 5% QSB: bread with 5% of unground quinoa seed.
